# Adaptation of *Hansenula polymorpha *to methanol: a transcriptome analysis

**DOI:** 10.1186/1471-2164-11-1

**Published:** 2010-01-04

**Authors:** Tim van Zutphen, Richard JS Baerends, Kim A Susanna, Anne de Jong, Oscar P Kuipers, Marten Veenhuis, Ida J van der Klei

**Affiliations:** 1 Molecular Cell Biology, University of Groningen, P.O. Box 14, 9750 AA Haren, the Netherlands; 2 Molecular Genetics, University of Groningen, P.O. Box 14, 9750 AA Haren, the Netherlands; 3 Kluyver Centre for Genomics of Industrial Fermentation, P.O. Box 5057, 2600 GA Delft, the Netherlands

## Abstract

**Background:**

Methylotrophic yeast species (e.g. *Hansenula polymorpha, Pichia pastoris*) can grow on methanol as sole source of carbon and energy. These organisms are important cell factories for the production of recombinant proteins, but are also used in fundamental research as model organisms to study peroxisome biology. During exponential growth on glucose, cells of *H. polymorpha *typically contain a single, small peroxisome that is redundant for growth while on methanol multiple, enlarged peroxisomes are present. These organelles are crucial to support growth on methanol, as they contain key enzymes of methanol metabolism.

In this study, changes in the transcriptional profiles during adaptation of *H. polymorpha *cells from glucose- to methanol-containing media were investigated using DNA-microarray analyses.

**Results:**

Two hours after the shift of cells from glucose to methanol nearly 20% (1184 genes) of the approximately 6000 annotated *H. polymorpha *genes were significantly upregulated with at least a two-fold differential expression. Highest upregulation (> 300-fold) was observed for the genes encoding the transcription factor Mpp1 and formate dehydrogenase, an enzyme of the methanol dissimilation pathway. Upregulated genes also included genes encoding other enzymes of methanol metabolism as well as of peroxisomal β-oxidation.

A moderate increase in transcriptional levels (up to 4-fold) was observed for several *PEX *genes, which are involved in peroxisome biogenesis. Only *PEX11 *and *PEX32 *were higher upregulated. In addition, an increase was observed in expression of the several *ATG *genes, which encode proteins involved in autophagy and autophagy processes. The strongest upregulation was observed for *ATG8 *and *ATG11*.

Approximately 20% (1246 genes) of the genes were downregulated. These included glycolytic genes as well as genes involved in transcription and translation.

**Conclusion:**

Transcriptional profiling of *H. polymorpha *cells shifted from glucose to methanol showed the expected downregulation of glycolytic genes together with upregulation of the methanol utilisation pathway. This serves as a confirmation and validation of the array data obtained. Consistent with this, also various *PEX *genes were upregulated. The strong upregulation of *ATG *genes is possibly due to induction of autophagy processes related to remodeling of the cell architecture required to support growth on methanol. These processes may also be responsible for the enhanced peroxisomal β-oxidation, as autophagy leads to recycling of membrane lipids. The prominent downregulation of transcription and translation may be explained by the reduced growth rate on methanol (t_d _glucose 1 h vs t_d _methanol 4.5 h).

## Background

*Hansenula polymorpha *is an important cell factory for the production of pharmaceutical proteins [1]. Moreover, it is extensively used in fundamental research aiming at understanding the molecular principles of peroxisome biology [2].

As cell factory, *H. polymorpha *has several important advantages. First, it contains very strong and inducible promoters derived from the methanol metabolism pathway. Also, the organism is thermotolerant (it can grow at high temperatures up to 48°C, [3]) and tolerates various environmental stresses. *H. polymorpha *does not hyperglycosylate secreted proteins, which often is a problem in heterologous protein production in *S. cerevisiae*.

In *H. polymorpha *peroxisomes massively develop during growth on methanol as sole source of carbon and energy. Methanol is oxidized by the enzyme alcohol oxidase (AOX), which is localized in peroxisomes together with catalase and dihydroxyacetone synthase (DHAS), the first enzyme of the assimilation pathway. Peroxisomes are not required for primary metabolism when cells are grown on glucose. Moreover, glucose represses the key enzymes of methanol metabolism AOX and DHAS. Therefore, during growth on glucose *H. polymorpha *cells contain only a single, small peroxisome. Upon a shift to methanol media, the enzymes of methanol metabolism are induced paralleled by an increase in peroxisome size and abundance. The initial small peroxisome serves as the target organelle for the enzymes of methanol metabolism and proliferates by fission [4]. Ultimately, in exponentially growing cells, each cell contains several enlarged peroxisomes [5].

A wealth of information is now available of individual genes encoding enzymes of the methanol metabolism as well as on genes involved in peroxisome formation (*PEX *genes). However, genomics approaches in *H. polymorpha *are still rare.

We used genome-wide transcriptional profiling to dissect the initial events accompanying the adaptation of glucose grown *H. polymorpha *cells to methanol metabolism. This will gain information on the induction and repression of metabolic genes as well as on non-metabolic genes, including *PEX *genes.

## Results and discussion

All experiments described in this paper were performed in batch cultures. We chose not to grow the cells in carbon-limited chemostats, as glucose-limitation results in derepression of genes involved in methanol metabolism [6].

**Figure 1:**
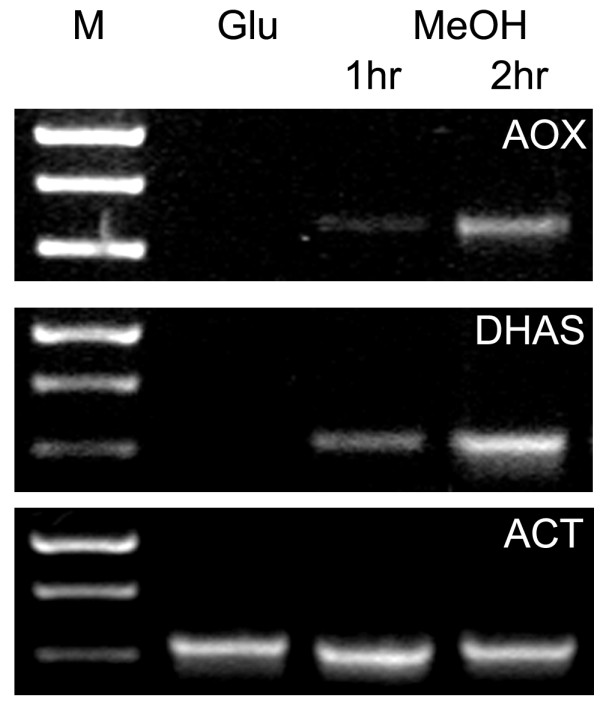
**Transcript level of *AOX *and *DHAS***. RT-PCR was performed on RNA samples using *AOX *and *DHAS *primers from glucose containing precultures (OD 2,3), and cultures shifted for 1 or 2 hrs to methanol medium. As loading control transcript levels of actin were analysed.

*H. polymorpha *cells were extensively pre-cultivated in batch cultures on mineral media supplemented with glucose as sole carbon source in order to fully repress the enzymes of methanol metabolism. Glucose cultures in the late exponential growth phase were transferred to fresh mineral medium containing methanol as sole carbon and energy source. As shown in figure 1, RT-PCR indicated that the inoculum cells (from the glucose batch culture at the late exponential growth phase, OD_660 nm _2.3) did not contain transcript of alcohol oxidase (AOX) or dihydroxyacetone synthase (DHAS), key enzymes of methanol metabolism. However, two hours after the shift to medium containing methanol, mRNAs of both genes were readily detected, a time-point which has also been identified as threshold for the detection of first AOX enzyme activity [5]. Therefore, 2 hours incubation on methanol was selected as sampling point of cells for transcriptome analysis.

Replicates were obtained by growing 4 independent cultures on glucose that were independently transferred to fresh media containing methanol. Of each culture, mRNA isolated from the glucose and the methanol sample was labeled (and dye-swapped) and hybridized on two arrays per culture. In addition, as a control *AOX *transcript levels of these samples were determined by RT-PCR, confirming the absence of transcript in the glucose-grown pre-cultures and the presence of *AOX *transcript after 2 hours incubation (data not shown).

### Overview of the DNA microarray data

The DNA microarray analyses data were analyzed to generate the ratio between the transcripts on methanol and glucose for each gene to identify any differential expression and to determine the p-value to assess the significance and the A-value to check the intensity of the signals. [Additional file 1: Supplemental table S1] presents an overview of the array results. Of the nearly 6000 annotated *H. polymorpha *genes that are listed, approximately 20% (1184 genes) are upregulated, while another 20% (1246 genes) are downregulated with at least a two-fold differential expression, meeting the significance and signal intensity criteria.

Of the upregulated genes, 13 are more than 100 times upregulated, 192 genes show a 10-100-fold upregulation, 156 genes increase between 5 and 10-fold and the remaining 823 genes are less than 5-fold upregulated. Highest upregulated are the central methanol metabolism regulator *MPP1 *(394-fold, Hp27g360, see below) and the gene encoding formate dehydrogenase (347-fold), required in methanol catabolism. Also the other components of the methanol metabolic pathway are highly upregulated. Moreover, *CRC1 *is highly induced, encoding a mitochondrial inner membrane carnitine transporter, which is required for the transport of acetyl-CoA from peroxisomes to mitochondria during fatty acid beta-oxidation (111-fold). In line with *CRC1*, also the genes involved in the beta-oxidation of fatty acids are overrepresented among the highly upregulated genes (for details see below). Furthermore, approximately 13% of the more than 10-fold upregulated genes is involved in transport. The upregulation of hexose transporters may be important for the uptake of the residual glucose that was present in the inoculum. Of the downregulated genes, the highest fold downregulation (65-fold) is observed for Hp24g956, encoding a protein with strong similarity to Sik1p of *S. cerevisiae*, which is involved in pre-rRNA processing. This predicted function is consistent with the general trend among the downregulated genes, since of the 179 genes that are over 10-fold downregulated, nearly 50% code for products that function in either transcription or translation processes. Of the other downregulated genes, 269 show a 5- to 10-fold downregulation. Of these, nearly 40% encode proteins involved in transcription and translation. The other 789 genes are less than 5-fold reduced in transcript levels.

### Functional overview DNA microarray data - FUNCATS

**Figure 2:**
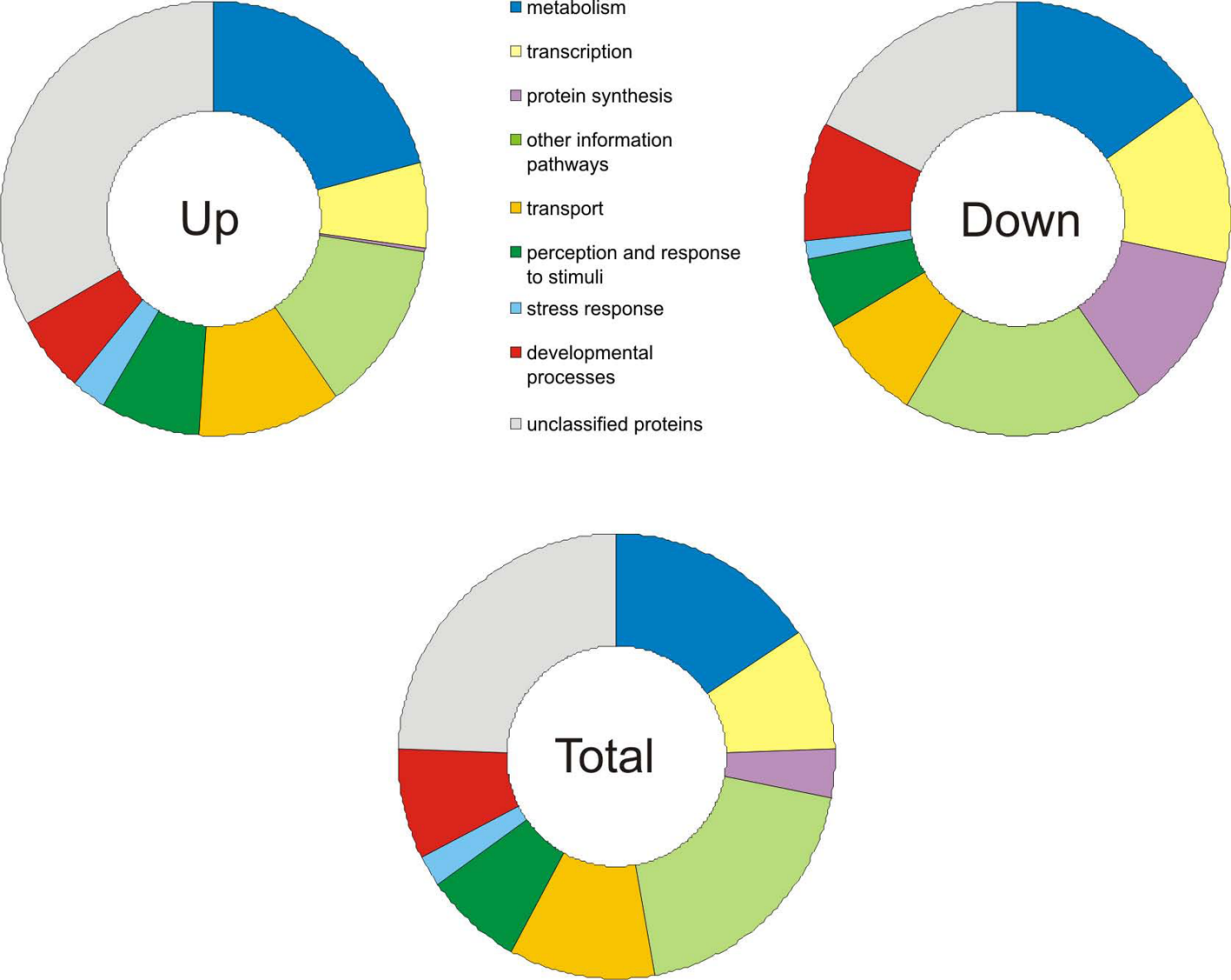
**FUNCATS**. Representation of functional groups among up- and downregulated genes is shown in a diagram. For comparison, a similar diagram is made for the total number of genes in *Hansenula polymorpha *(up-, down- and non-regulated genes). Genes are classified in one or multiple groups based on the Functional Catalogue.

To obtain an overview of the functions of the differentially expressed genes, these were categorized according to the Functional Catalogue, FUNCAT [7,8]. In this system, each gene is classified in one or more groups, depending on its function. The number of genes in each category is shown as the percentage of the total number of up- or downregulated genes in the diagrams shown in figure 2. For comparison, a diagram showing the contribution of each functional category to the total number of genes in *H. polymorpha *is included. To construct this diagram, all known *H. polymorpha *genes are used; both up- and downregulated genes as well as non-regulated genes.

As expected, genes involved in metabolic pathways strongly contribute to both the up- and downregulated genes (20% and 15.5%, respectively), reflecting the large-scale adaptations accompanying the shift from glucose to methanol. However, metabolism is a large group also in the total genome and the contribution in percentages does not reflect the nature of the metabolic pathways that are regulated (see below).

In contrast to metabolism, some other functional categories display a difference in contribution to the upregulated compared to the downregulated genes. One such functional category is the group of protein synthesis genes, which is almost absent among upregulated genes (0.25%), while it composes a large portion of the downregulated genes (12.1%). Of the total genome of *H. polymorpha *only approximately 4% is involved in protein synthesis, reflecting the considerable effect of a shift to methanol on protein synthesis. In addition, also the group of genes involved in transcription is more predominant among downregulated genes (12.7% versus 6.3% of the upregulated genes and an intermediate 9% of the total genome). In concurrence with the trend of genes related to transcription and translation, also genes related to nucleotide biosynthesis are mostly downregulated (42 of 51 genes), yet genes involved in amino acid biosynthesis show a less clear trend (30 down-, 9 upregulated, 54 not differentially expressed). The observed downregulation of major anabolic processes most likely is related to the reduction in doubling time (t_d _of cells on methanol relative to growth on glucose (t_d _methanol = 4.5 h, t_d _glucose = 1 h) and may reflect the accompanying decrease in DNA replication, RNA transcription and translation.

Stress response genes form only relatively small categories among both the upregulated genes relative to the downregulated genes (2.9% vs 1.6%). Based on the Functional Catalogue, only 185 of the nearly 6000 annotated *H. polymorpha *genes are indicated as stress response genes. However, based on several studies by Gasch in *Saccharomyces cerevisiae *[9-11], many more genes could contribute to the cellular stress response. Hence, most likely also genes classified in other groups play a role in coping with stress accompanying a shift in cultivation conditions. Thus, the contribution of the stress response to the total differential expression in *H. polymorpha *upon transfer to methanol medium is probably larger than the observed 2.9% upregulation and 1.6% downregulation.

A last remarkable group in the Functional Catalogue diagram is the category of unclassified proteins, showing that 33% of the upregulated genes and 17.9% of the downregulated genes are thus far not experimentally characterized, relative to 25% of the genes of the total genome of *H. polymorpha*. This observation suggests that our current knowledge on adaptation to methanol is far from complete.

### Metabolic pathways - upregulation of methanol metabolism

**Figure 3:**
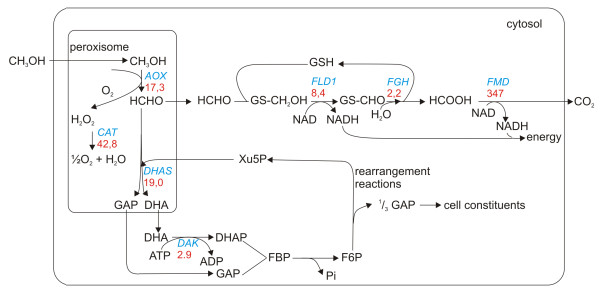
**Methanol metabolism**. Overview of the methanol metabolism in *H*. *polymorpha*. The fold upregulation of the indicated genes is shown in red. GAP: glyceraldehyde-3-phosphate; DHA: dihydroxyacetone; DHAP: dihydroxyacetone phosphate; FBP: fructose bisphosphate; F6P: fructose-6-phosphate; XU5P: xylulose-5-phosphate; GSH: reduced glutathione; GS-CH_2_OH: S-hydroxymethylglutathione; GS-CHO: S-formylglutathione. *AOX*: alcohol oxidase; *CAT*: catalase; *DHAS*: dihydroxyacetone synthase; *DAK*: dihydroxyacetone kinase; *FLD*: formaldehyde dehydrogenase; *FGH*: S-formyl glutathione hydrolase and *FMD*: formate dehydrogenase.

As expected, genes involved in methanol metabolism are highly upregulated. In figure 3 an overview of the methanol metabolism, including the microarray data, is presented [2]. In peroxisomes, methanol is oxidized to formaldehyde and hydrogen peroxide by alcohol oxidase (AOX), which is 17.3 times upregulated at the transcriptional level. The hydrogen peroxide is detoxified by catalase (CAT) to water and oxygen (42.8-fold upregulated). Formaldehyde can be further metabolized via two different routes: 1) dissimilation via formaldehyde dehydrogenase (FLD1, 8.4-fold up), S-formyl glutathione hydrolase (FGH, 2.2-fold up) and formate dehydrogenase (FMD, 347-fold up) to CO_2_, generating NADH and CO_2 _or 2) assimilation via the peroxisome-borne enzyme dihydroxyacetone synthase (DHAS, 19,0-fold up) to generate cell constituents. DHAS is part of the xylulose-5-phosphate cycle and catalyzes the formation of two C3-molecules (dihydroxyacetone and glyceraldehyde-3-phosphate) from one C1 (formaldehyde) and one C5 compound (xylulose-5-phosphate) [2].

Promoter studies in *Candida boidinii *using phosphatase as a reporter enzyme revealed that upon a shift to methanol medium *FMD *was induced first, followed by *DHAS *and even later *AOX *[12]. The early induction of *FMD *(347-fold up 2 hours after the shift to methanol medium) relative to *AOX *and *DHAS *(17.3-, 19-fold up respectively) suggests that a similar induction pattern may exist in *H. polymorpha*. The differences in induction of the genes 2 hours after the shift to methanol medium does not reflect the ultimate protein levels in the cells, as AOX and DHAS are the major protein bands in extracts prepared from methanol grown *H. polymorpha *cells [12,13].

### PEX genes

*PEX *genes control the development and function of a specialized class of organelles, the peroxisomes. Most of the *PEX *genes showed a moderate induction upon the shift to methanol (up to 4-fold; Table 1). This is in line with earlier studies of *S. cerevisiae *cells that were shifted from glucose to the peroxisome-inducing carbon source oleate [14,15]. Of the *PEX *genes involved in import of peroxisomal matrix enzymes (AOX, DHAS and CAT), the highest upregulation was observed for *PEX1, PEX4, PEX5, PEX13, PEX14 *and *PEX26*, which all encode key components PTS1 protein import machinery [16]. Highest upregulation was observed for *PEX11 *(4.7-fold) and *PEX32 *(4.8-fold). Pex11p, together with Pex25p and Pex11cp, are the three members of the Pex11p protein family in *H. polymorpha *[16]. These proteins are implicated in regulating peroxisome proliferation. Also in bakers' yeast cells shifted from glucose to oleic acid medium *PEX11 *increased by far the most [14].

**Table 1:** Expression changes of *PEX *genes

*PEX *genes		Ratio
Hp46g103	*PEX1*	2.9

Hp24g603	*PEX2*	1.7

Hp47g896	*PEX3*	1.5

Hp13g30	*PEX4*	3.1

Hp28g69	*PEX5*	3.3

Hp33g316	*PEX6*	1.6

Hp15g912	*PEX7*	1.8

Hp27g144	*PEX8*	1.7

Hp6g229	*PEX10*	1.6

Hp24g562	*PEX11*	4.7

Hp5g555	*PEX11C*	-1.6

Hp39g539	*PEX12*	2.6

Hp32g232	*PEX13*	3

Hp24g522	*PEX14*	3.5

Hp14g184	*PEX17*	2.1

Hp9g314	*PEX19*	1.1

Hp11g43	*PEX20*	1

Hp37g108	*PEX22*	-1.3

Hp39g248	*PEX23*	1.2

Hp25g249	*PEX23-like*	-1.2

Hp47g626	*PEX24*	2.9

Hp16g88	*PEX25*	2.2

Hp15g17	*PEX26*	3.6

Hp29g7	*PEX29*	-1.2

Hp27g236	*PEX32*	4.8

All genes shown have a p-value below 0.05. Negative values indicate downregulation on methanol, positive values indicate upregulation on methanol

*H. polymorpha PEX25 *was upregulated 2.2-fold, whereas *PEX11C*, whose function is still unknown, showed a 1.6-fold downregulation [16].

**Figure 4:**
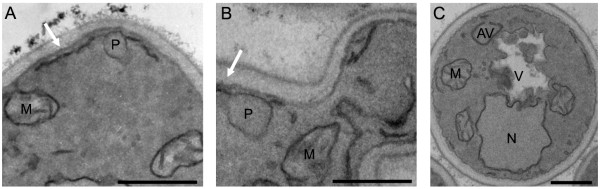
**Ultrastructural analysis of the adaptation of cells to methanol**. Glucose-grown *H. polymorpha *WT cells were extensively analysed at different time-points after the shift to methanol by electron microscopy of KMnO_4_-fixed samples. (A) Control glucose-grown cell and (B) after 2 h of incubation in the presence of methanol. A clear increase in peroxisome size was observed, cross-sections of representative cells are depicted. Note the association of the organelles with strands of ER (indicated by arrow). (C) 2 hours after the shift a clear increase was observed in large vacuolar autophagic bodies, indicative of induction of autophagy. N - Nucleus, M - Mitochondrion, V - Vacuole, AV - Autophagosome. The bar represents 0.5 μm.

Pex32p is a member of the Pex23 protein family, a group of membrane proteins with unknown function [16]. *Y. lipolytica pex23 *mutants cannot grow on oleate and partially mislocalize peroxisomal proteins to the cytosol, suggesting a role in matrix protein import. In contrast however, *S. cerevisiae *Pex23p, Pex31p and Pex32p are not required for protein import but play a role in peroxisome proliferation. Where ScPex23p appears to be a positive regulator of peroxisome size, ScPex31p and ScPex32p have been suggested to negatively regulate this process. The function of *H. polymorpha *Pex32p is not yet known. Based on our current study this protein may be, together with Pex11p, responsible for the initial increase in size of the peroxisomes, as was observed by detailed ultrastructural analysis (figure 4) and in concurrence with earlier findings [5]. The relatively high induction of this peroxin makes it an interesting candidate for further study in *H. polymorpha*.

### Metabolic pathways - downregulation of glucose utilisation

As depicted in Table 2, the majority of the genes involved in glycolysis are downregulated (-1.2 to -5.7). Since the genes are listed in order of appearance in the pathway, it is evident that the strongest downregulation is observed in the steps before fructose 1,6 bisphosphate aldolase. This corresponds with the fact that the products of methanol metabolism, dihydroxyacetone and glyceraldehyde 3 phosphate, enter the glycolytic pathway directly after this step, so the subsequent enzymes are still required for progression with the pathway towards the TCA cycle. The observed mild decrease in expression of their encoding genes can be attributed to the reduction in the substrate flow, when switching from glucose to methanol utilisation. However, it should be noted that the enzymes of the final part of glycolysis in majority also function in the direction of gluconeogenesis, by catalyzing the reverse reactions. Finally, the upregulation of the gene encoding fructose 1,6 bisphosphate aldolase which, on methanol, catalyzes the formation of fructose 1,6 bisphosphate from dihydroxyacetone and glyceraldehyde-3-phosphate, reflects an increase of this reaction, which has been shown to be important in the rearrangement reactions to replenish the cell with xylulose-5-phosphate to the downstream reactions in methanol metabolism [2].

**Table 2:** Expression changes of glycolysis and gluconeogenesis genes

Gene	Function	Ratio
**Glycolysis**		

Hp25g374	Hexokinase	-5.7

Hp24g239	Glucokinase	-2.3

Hp33g380	Glucose-6-phosphate isomerase	-1.7

Hp1g417	Phosphofructokinase alpha subunit	-2.9

Hp39g214	Phosphofructokinase beta subunit	-2

Hp47g1019	Fructose 1,6-bisphosphate aldolase	2.1

Hp16g222	Triosephosphate isomerase	-1.5

Hp25g306	Glyceraldehyde-3-phosphate dehydrogenase	-1.4

Hp26g207	3-phosphoglycerate kinase	-1.3

Hp37g8	Phosphoglycerate mutase	1.2*

Hp27g405	Phosphopyruvate hydratase (enolase)	-1.7

Hp39g227	Pyruvate kinase	-1.2

Hp6g262	Pyruvate dehydrogenase, alpha subunit	-1.3

Hp37g184	Pyruvate dehydrogenase, beta subunit	-1.8

**Gluconeogenesis**		

Hp18g102	Pyruvate carboxylase	1.9

Hp5g547	Phosphoenolpyruvate carboxykinase	8.9

Hp46g88	Fructose 1,6 bisphosphatase	4.4

* With the exception of the one marked with an asterisk, all genes shown have a p-value below 0.05.

### Regulatory networks

Accompanying the changes in expression of many metabolic genes, also changes in the underlying regulatory networks are expected. In addition to global changes, the DNA microarray data reflect the initial adaptation to methanol and thus enable the investigation of more specific changes resulting in activation of methanol-dependent genes or in repression of glucose-dependent genes.

Among the upregulated genes, 49 are involved in regulation of transcription. Most of these encode general transcription factors or transcription factors which have not been linked to a specific process. Regulators involved in stress response (6), glucose sensing/derepression (4), and peroxisome-related pathways (4) are overrepresented, as is expected due to the change in carbon source.

The expression of genes coding for peroxisome assembly and function is controlled by a transcriptional regulatory network, which has been studied extensively in *S. cerevisiae *in response to oleate [17-19]. The Oaf1-Pip2 complex plays a prominent role, although the putative *H. polymorpha *homolog of *PIP2 *is only moderately upregulated during adaptation to methanol. However the homolog of *ADR1 *(23.7-fold), a second activator of oleate-responsive genes is strongly upregulated in *H. polymorpha *and is also involved in regulation of the response to both oleate and methanol in *P. pastoris *(named *MXR1*; [20]. Virtually all known targets of Adr1 and its co-regulator Cat8 were indeed also upregulated in *H. polymorpha*, suggesting an important role in regulation of methanol metabolism, while most probably also activation by Snf1 is initiated after the shift, since this global regulator is crucial for growth on non-fermentable carbon sources [21,22]. Mpp1, another transcriptional regulator of methanol metabolism in *H. polymorpha*, is encoded by the strongest upregulated gene of this study (Hp27g360, 394-fold up), thus suggesting it is a master regulator of methanol-responsive genes [23].

Swi1 and Snf2 also belong to a regulatory complex involved in gene expression of methanol-related genes as well as peroxisomal assembly, however their encoding genes were not induced in the early adaptation phase to methanol [24].

Among the downregulated genes, the decreased transcription of *RAP1 *(Hp16g154, -3.1) is remarkable. This transcriptional regulator is known to activate transcription of genes encoding ribosomal proteins [25,26] and its downregulation is consistent with the observed massive decrease in transcripts for ribosomal proteins. Interestingly, this gene is also shown to be repressed during the environmental stress response in *S. cerevisiae *as described by Gasch *et al*., [9].

### Autophagy

Adaptation of *H. polymorpha *cells to methanol requires a major rearrangement of the cellular architecture. The finding that most *ATG *genes, which are involved in autophagy and autophagy related processes [27], as well as several proteasomal genes are upregulated (18 up vs 2 down), suggests that cellular reorganisation requires massive degradation of cellular components. Interestingly, the highest upregulation is observed for *ATG8 *and *ATG11 *(Table 3). Atg8 has a prominent role in various selective and non-selective macroautophagic processes, whereas Atg11 is specifically involved in selective macroautophagy [28,29]. The function of HpAtg19-like, of which the encoding gene is also upregulated, is not known. However, HpAtg19-like is not involved in selective degradation of peroxisomes (unpublished data). Remarkably only *ATG25 *is significantly downregulated. Atg25 is involved in selective peroxisome degradation by macropexophagy, but not in microautophagy [29].

**Table 3:** Expression changes of *ATG *genes

Autophagy-related genes		Ratio
Hp24g929	*ATG1*	3.7

Hp15g1008	*ATG2*	2.6

Hp42g317	*ATG3*	3.9

Hp24g999	*ATG4*	2.3

Hp47g352	*ATG5*	1.8

Hp24g284	*ATG6*	3.7

Hp19g8	*ATG7*	2.2

Hp48g37bm	*ATG8*	6.2

Hp16g127	*ATG9*	1.6

Hp24g546m	*ATG10*	2.5

Hp25g507	*ATG11*	5.2

Hp33g43	*ATG12*	2.3

Hp19g348	*ATG13*	1.6

Hp47g589	*ATG15*	2.2

Hp24g680m	*ATG16*	1.7

Hp8g289	*ATG17*	1.1*

Hp25g289	*ATG18*	1.9

Hp13g64	*ATG19*-like	6.6

Hp16g331	*ATG20*	-1.1

Hp44g480	*ATG21*	1.8

Hp18g58	*ATG22*	1.7

Hp33g356	*ATG24*	2.8

Hp39g230	*ATG25*	-4.3

Hp15g447	*ATG26*	2.1

Hp39g339	*ATG27*	-1.7

Hp47g741	*ATG28*	1.8

Hp32g359	*ATG30*	4.1

*With the exception of the one marked with an asterisk, all genes shown have a p-value below 0.05.

Ultrastructural analysis of cells at different time-points after the shift indeed showed strong morphological evidence for increased autophagy, reflected in the massive uptake of cytoplasmic components in the vacuole (figure 4).

Recent findings showed the importance of autophagy during methanol adaptation of *P. pastoris*, not only for cell remodeling, but also to provide amino acids [30]. Consistent with these findings, we also observed that *H. polymorpha atg *mutants showed a slight delay in methanol adaptation (data not shown).

### β-oxidation of fatty acids

A significant upregulation of genes encoding proteins related to β-oxidation of fatty acids was observed [31,32]. This unexpected cluster is listed in Table 4. The regulation of these genes could be merely due to derepression as a result of decreasing glucose levels. However we consider this less likely since the observed ratio's and signals are quite substantial. Another explanation could be co-regulation of multiple peroxisomal pathways by common regulators. A third option is the increase in cellular fatty acid levels, the substrate for peroxisomal β-oxidation. This might originate from the observed autophagy, leading to recycling of intracellular membrane lipids.

**Table 4:** Expression changes of genes related to fatty acid β-oxidation

Gene	Function	Ratio
Hp8g534	Peroxisomal ABC-transporter sub-unit 1	8

Hp33g390	Peroxisomal ABC-transporter sub-unit 2	6.4

Hp44g158	Adenine nucleotide transporter	3

Hp33g132	Fatty-acyl coenzyme A oxidase	14

Hp8g261	Multifunctional enzyme	21.6

Hp24g1381	3-ketoacyl-CoA thiolase	16.3

Hp27g292	Catalase	42.8

Hp29g305	Isocitrate lyase	30.8

Hp43g61	Malate synthase	8.8

Hp36g14	Isocitrate dehydrogenase	27.7

Hp47g959	Carnitine acetyl-CoA transferase	41.6

Hp39g121	Carnitine acetyltransferase, *YAT1*	47.1

Hp8g466	Carnitine acetyltransferase, *YAT2*	36

Hp15g677	Mitochondrial carnitine/acyl carnitine carrier	111.3

All genes shown have a p-value below 0.05.

### Mitochondria

**Figure 5:**
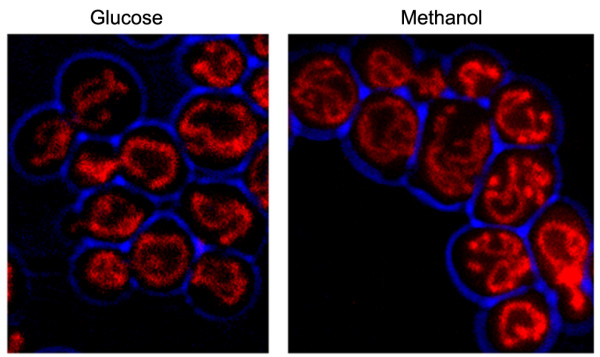
**Fluorescence microscopy of mitochondria**. Fluorescence microscopy images demonstrating the increase in mitochondrial volume fractions in cells after 2 hours of incubation on methanol (B), relative to those of the glucose inoculum cells (A). Mitochondria are marked by MitoTracker Orange.

Remarkably, the shift of cells from glucose to methanol is associated with a significant increase in mitochondrial volume fractions (figure 5). Several functional links exist between peroxisomes and mitochondria, both for metabolic pathways and for biogenesis of both organelles [33,4]. Of the genes involved in mitochondrial function or assembly 110 are down- and 67 are upregulated. Genes coding for components of the Dnm1-dependent fission machinery of both organelles are not differentially expressed [4]. Similar to all downregulated genes, of the mitochondrial downregulated genes almost 50% is involved in transcription and translation processes. In addition the genes coding for TOM and TIM protein import complexes are also mostly down-regulated. Genes involved in FeS cluster, heme biosynthesis and cytochrome c assembly are overrepresented among upregulated mitochondrial genes (9-fold), in agreement with the prominent role for mitochondria as the sole site of ATP generation during methanol-metabolism [2]. Heme is also the co-factor of peroxisomal catalase which is highly induced. FeS cluster formation is also coupled to the glutathione-based redox regulation system via *GRX5 *[34].

### Reactive oxygen species

Although mitochondria were long considered as the main source of reactive oxygen species (ROS), also peroxisomes actually defined as organelles that harbour H_2_O_2_-producing enzymes as well as catalase are now recognized as a significant contributor to ROS production [2,35,36]. Besides catalase (42.8-fold up), peroxisomes also contain the peroxiredoxin Pmp20, that is 11-fold upregulated, both indicative of an increase in peroxisomal ROS production [37,38].

We also observed an increase of several pivotal genes involved in cytosolic and mitochondrial ROS detoxification; like the superoxide dismutase *SOD1 *(4.3-fold), the glutathione transferase *GTO2 *(2.6-fold), the glutathione reductase *GPX3 *(8-fold), the glutaredoxin *GRX5 *(2.8-fold) and the thioredoxin peroxidase *TSA1 *(47.2-fold). The remaining members of the glutathione- and TRX-based system are not differentially expressed (8 other genes found), except for the thioredoxin reductase *TRR1 *(10.4-fold). Induction of these key enzymes involved in sustaining the redox balance of the cytosol, suggests that methanol-metabolism also results in enhanced cytosolic ROS levels, which may originate from peroxisomal metabolism.

### Comparison with other DNA microarray analyses

Back in 1996, the proof of principle for the use of DNA microarray technology to investigate transcriptional changes was shown for *S. cerevisiae *[39]. Since then, DNA microarray analysis has become a regular, well-established tool, facilitated by the now commercial available array slides. For many other yeast species however, thusfar the usage of DNA microarray analysis is still rather limited. Only recently, species-specific DNA microarray studies have been presented for e.g. *Debaryomyces hansenii *[40] and *P. pastoris *[41]. For *H. polymorpha*, Oh *et al.*, [42] presented a partial, DNA microarray study, followed by a full DNA microarray analysis describing the transcriptional response of *H. polymorpha *to exposure to cadmium [43]. However, this study was not related to the metabolic and architectural alterations associated with a change in cellular metabolism.

Smith *et al*. [15,18] published two studies in which bakers' yeast was shifted to oleate. The first study focused mainly on transcriptional changes of genes encoding peroxisomal proteins and peroxins, the second one on the regulatory network coordinating the response to oleate.

The current study is the first in which arrays were used to study the shift from glucose to methanol in the yeast *H. polymorpha*. Sauer *et al*. [44] was the first to publish transcriptional profiling of the methylotrophic yeast *P. pastoris*, also upon a shift to methanol. However, in that study, yeast cells were transferred from glycerol to methanol and heterologous hybridisation onto *S. cerevisiae *DNA-microarrays was performed. Still, the same trend in regulated functional groups was observed. Also, similar effects were reported on carbohydrate metabolism and the regulation of ribosomal genes. Although some of the data obtained for *H. polymorpha *are comparable with those obtained for *P. pastoris*, a significant drawback of the latter study is the absence of *P. pastoris *specific or methylotrophic yeast specific genes on the DNA-microarray slides. There is indeed a substantial amount of methanol-responsive *H. polymorpha *genes found in our study which were not observed in *P. pastoris *using *S. cerevisiae *microarrays (± 450 upregulated, ± 350 downregulated) [Additional file 2: Supplemental Table S2].

## Conclusions

The current DNA microarray study revealed the expected repression of genes involved in glucose metabolism concomitant with the induction of the genes of methanol metabolism in response of a shift of *H. polymorpha *cells from glucose to methanol. Also genes involved in peroxisome biogenesis (*PEX *genes) are upregulated, with *PEX32 *being the most strongly upregulated *PEX *gene. A surprising finding was the upregulation of autophagy- and of beta-oxidation-genes. The first can likely be explained by the need for cellular reorganisations and is confirmed by electron microscopy studies showing active uptake of cytoplasmic components in the vacuoles of the cells. The induction of beta-oxidation is thought to be a consequence of the cellular reorganisations and thereby the high turnover of lipids, serving as substrates for beta-oxidation. A final interesting but not yet uncovered group consists of the 33% of the upregulated genes that have no known function. These genes reflect a large potential of *H. polymorpha *or methylotrophic yeast specific genes with a specialized role in adaptations to growth on methanol as the sole source of carbon and can form interesting targets for future research.

## Methods

### *H. polymorpha *microarray probe design

*H. polymorpha *open reading frame sequences were collected from the *H. polymorpha *genome database (Rhein Biotech, unpublished; [7] and NCBI [45-47]. For the genes on contigs 1 - 48, the annotation was based on the information from RheinBiotech and Ramezani-Rad *et al*. [7]. The additional NCBI sequences were listed as Hp50 and Hp51 numbers. The annotation of these genes was as described on NCBI (Hp50s) or by manual blast search (Hp51s). All Hp sequences were applied for design of oligonucleotide probes using OLIGOARRAY v2.1 [48] with the following oligonucleotide primer design parameters: a length of 58-60 nucleotides, a melting temperature (Tm) of at least 80°C, a G/C-content of 34-52% and a maximum Tm of secondary structures and cross-hybridisations of 68°C. Oligo's were designed within the 3'-regions of the ORFs (setting: maximum distance between the 5' end of the oligo and the 3' end of the input sequence: 600-nt) to minimise including intron-sequences, since these were not discarded in the input ORF sequences. Paralogous sequences were removed during the final analysis of the design using blastN. Of the 6,248 oligo-probes, 6,002 are from the annotated genes of *H. polymorpha *and 23 probes are from heterologous genes of specific research interest (data not shown). The remaining 223 probes include positive and negative controls. Subsequently, the oligo-set was printed twice in each of the 8 arrays per slide (8-plex format) using Agilent's SurePrint technology (*in situ *synthesis; via eArray 4.0-website; Agilent Technologies Netherlands B.V., Amstelveen, the Netherlands).

### Organisms & Growth

*H. polymorpha *strain NCYC 495 *leu*^- ^was grown in batch cultures on mineral medium containing either 0.5% glucose or 0.5% methanol as carbon source and 0.25% ammonium sulphate as nitrogen source [49,50]. For transcriptome analysis, cells were extensively pre-cultivated in batch cultures on glucose at 37°C prior to a shift to fresh media containing methanol as sole carbon source. Four independent glucose-grown cultures were used to inoculate fresh medium containing methanol as well as for taking samples for RNA isolation. The methanol cultures were grown for two hours, followed by RNA isolation.

### RNA isolation

Samples were harvested by freezing them directly in liquid nitrogen, followed by thawing on ice, centrifugation (5.000 G, 4 min, 0°C) and taken up in AE-buffer (50 mM sodium-acetate 10 mM EDTA pH 5.0). 1 volume acid-phenol chloroform isoamylalcohol (125:24:1 pH 4.5, Ambion, Austin USA) and 0.5% SDS was added, and incubated at 65°C for 5 min followed by 15 min at -80°C. After centrifugation (15 min 13.000 G), the upper phase was mixed with 0.5 volume acid phenol/chloroform, centrifuged (4 min 13.000 G) and mixed with 0.5 volume chloroform. The upper phase was used for RNA isolation using column purification according to the manufacturers' instructions (Nucleospin RNA II, Macherey-Nagel, Düren Germany). The Agilent Bioanalyzer 2100 with RNA 600 Nano chips was used to analyze the quality and integrity of the RNA samples.

### Biochemical methods

Transcript levels of methanol-related genes (*AOX *and *DHAS*), using those of actin as control, were determined by semi-quantitative RT-PCR, using actin as loading control (Ready-to-go RT-PCR beads, GE Healthcare, Little Chalfont UK).

Primers:

AOX-forw: CGTGAGAACAGTGCCAATGAAG

AOX-rev: TCACCGATGGTCAATGCAGTAG

DHAS-forw: GCAGGACGTGTACGACTTCTTC

DHAS-rev: GTAGGACGCCGTAGCGTATCTC

Act1-forw: GGTCATTGATAACGGATCCGG

Act1-rev: CACTTGTGGTGGACAATGGATGG

Cell lysates were essentially obtained as described [51], for subsequent AOX activity measurements as described [52].

### DNA microarray analysis - labeling, hybridisation, washing and scanning

Using the Low RNA input linear Amplification kit from Agilent, cDNA was generated based on 500 ng of each isolated mRNA sample. Next, cRNA was made using Cy3-CTP or Cy5-CTP incorporation for labeling purposes. For each original mRNA, a portion of Cy3 and Cy5 labeled cRNA was generated. The concentration and incorporation of the cRNA and the dyes are measured using Nanodrop. For each biological replicate, 300 ng Cy3 labeled glucose culture originating cRNA was used for hybridisation against 300 ng Cy5 labeled methanol culture originating cRNA and vice versa for the dye-swap, resulting in 8 hybridisations in total. Hybridisation, washing and scanning were performed according to the Agilent 'Two-color Microarray-based gene expression analysis protocol' (version 5.5, February 2007) by ServiceXS (Leiden, The Netherlands).

### Data analysis - hybridisation ratio's, A-values and p-values

To extract the data from the scanned DNA-microarray slides, the feature extraction software version 9.5, Protocol GE2-v5_95_Feb07 from Agilent was used. For the background subtraction the option 'No background subtraction and spatial detrend' was used. For each spot, the ratio between the green and red processed signals was calculated, reflecting the ratio of gene expression on methanol overexpression on glucose. Next, the average ratio per gene was calculated based on the data of 16 spots (8 hybridisations, 2 spots per hybridisation). For reasons of clarity, genes with a ratio of <1 were expressed as -(1 divided by the ratio), thus reflecting the fold downregulation (e.g. -2 instead of 0.5). As a cut-off for differential gene expression, a threshold of (more than) 2-fold up- or downregulation was used, so >2 or <-2. To assess the significance of the data, p-values were computed using the paired-data program at the CyberT interface [53,54]. Genes were considered to be significantly regulated if they had a p-value below 0.05. In addition, average A-values for each gene were calculated as an indication for the intensity of the signals using A = 0.5*(log2Cy3+log2Cy5). An A-value of 6 was used as a lower limit for trustworthy signal intensity.

All data has been deposited to the NCBI Gene expression omnibus and is accessible under accession number GSE19036.

### Classification according to the Functional Catalogue

To show the main represented functions among up- and downregulated genes, *H. polymorpha *genes were ordered according to their Functional Catalogue (FunCat) as assigned by RheinBiotech. In the diagrams, the main groups of the hierarchical structure are shown as well as the subgroups 'transcription' and 'protein synthesis'[8]. The group 'subcellular localisation' was omitted, while 'control of cellular organisation', which is not in the FunCat structure, was included under 'developmental processes'. Genes can be present in more than one group.

### Analysis of metabolic routes using Biocyc

Changes in expression of metabolic pathway genes were investigated using the omics viewer at the Ecocyc website [55]. Since information on *H. polymorpha *is not included in this database, the genome of *Saccharomyces cerevisiae *S288C was used as a reference.

### Microscopy

Ultrathin sections of KMnO_4_-fixed cells were used for ultrastructural analysis as described [56]. Analysis of mitochondria was performed using confocal microscopy (Zeiss LSM510). Mitochondria were visualized using MitoTracker Orange (CMTMRos, Invitrogen) and visualization with excitation by a 543 nm Neon-laser (Lasos) and detection using a 565-615 band-pass emission filter.

## Authors' contributions

TvZ was involved in the annotation of the sequences used for the DNA microarray and performed the DNA microarray experiments. Furthermore, he has been active in data analysis and performed the additional experiments. RJSB performed the sequence annotation and designed and performed the DNA microarrays. He also has been involved in the data analysis. KAS has performed the DNA microarray analysis. AdJ has been involved in design of the DNA microarray slides. OPK supervised the array analysis. MV was involved in microscopy analysis and writing of the paper. IJvdK was general supervisor and involved in writing of the paper. All authors have read and approved the final manuscript.

## Supplementary Material

Additional file 1 **Supplemental Table S1**. Overview of complete array results. Table contains Ratio's, A-values and P-values from all genes. Click here for file

Additional file 2 **Supplemental Table S2**. All methanol-responsive genes (up- & down-regulated), which lack a homologue in *S. cerevisiae*. Click here for file

## References

[B1] Gellissen G Kunze G Gaillardin C Cregg JM Berardi E Veenhuis M Klei IJ van der New yeast expression platforms based on methylotrophic *Hansenula polymorpha *and *Pichia pastoris *and on dimorphic *Arxula Adeninivorans *and *Yarrowia lipolytica *- A comparison FEMS Yeast Res 2005 5 1079 1096 10.1016/j.femsyr.2005.06.004 16144775

[B2] Klei IJ Van der Yurimoto H Sakai Y Veenhuis M The significance of peroxisomes in methanol metabolism in methylotrophic yeast Biochim Biophys Acta 2006 1763 1453 1462 10.1016/j.bbamcr.2006.07.016 17023065

[B3] Ischuk OP Voronovsky AY Abbas CA Sibirny AA Construction of *Hansenula polymorpha *strains with improved thermotolerance Biotechnol Bioeng 2009 104 911 919 10.1002/bit.22457 19575437

[B4] Nagotu S Krikken AM Otzen M Kiel JAKW Veenhuis M Klei IJ van der Peroxisome fission in *Hansenula polymorpha *requires Mdv1 and Fis1, two proteins also involved in mitochondrial fission Traffic 2008 9 1471 1484 10.1111/j.1600-0854.2008.00772.x 18513378

[B5] Veenhuis M Keizer I Harder W Characterization of peroxisomes in glucosegrown *Hansenula polymorpha *and their development after the transfer of cells into methanol-containing media Arch Microbiol 1979 120 167 175 10.1007/BF00409104

[B6] Eggeling L Sahm H Derepression and Partial Insensitivity to Carbon Catabolite Regression of the Methanol Dissimilating Enzymes in *Hansenula polymorpha* Europ J Appl Microbiol Biotechnol 1978 5 197 202 10.1007/BF00579339

[B7] Ramezani-Rad M Hollenberg CP Lauber J Wedler H Griess E Wagner C Albermann K Hani J Piontek M Dahlems U Gellissen G The *Hansenula polymorpha *(strain CBS4732) genome sequencing analysis FEMS Yeast Res 2003 4 207 215 10.1016/S1567-1356(03)00125-9 14613885

[B8] Ruepp A Zollner A Maier D Albermann K Hani J Mokrejs M Tetko I Güldener U Mannhaupt G Münsterkötter M Mewes HW The FunCat, a functional annotation scheme for systematic classification of proteins from whole genomes Nucl Acid Res 2004 32 5539 5545 10.1093/nar/gkh894 PMC524302 15486203

[B9] Gasch AP Spellman PT Kao CM Carmel-Harel O Eisen MB Storz G Botstein D Brown PO Genomic expression programs in the response of yeast cell to environmental changes Mol Biol Cell 2000 11 4241 4257 11102521 10.1091/mbc.11.12.4241 PMC15070

[B10] Gasch AP Werner-Washburne M The genomics of yeast responses to environmental stress and starvation Funct Integr Genomics 2002 2 181 192 10.1007/s10142-002-0058-2 12192591

[B11] Gasch AP Comparative genomics of the environmental stress response in ascomycete fungi Yeast 2007 24 961 976 10.1002/yea.1512 17605132

[B12] Yurimoto H Komeda T Lim CR Nakagawa T Kondo K Kato N Sakai Y Regulation and evaluation of five methanol-inducible promoters in the methylotrophic yeast *Candida boidinii* Biochim Biophys Acta 2000 1493 56 63 10978507 10.1016/s0167-4781(00)00157-3

[B13] Roa M Blobel G Biosynthesis of peroxisomal enzymes in the methylotrophic yeast *Hansenula polymorpha* Proc Natl Acad Sci USA 1983 80 6872 6876 10.1073/pnas.80.22.6872 16593389 PMC390088

[B14] Kal JA van Zonneveld AJ Benes V Berg M van den Koerkamp MG Albermann K Strack N Ruijter JM Richter A Dujon B Ansorge W Tabak HF Dynamics of gene expression revealed by comparison of serial analysis of gene expression transcript profiles from yeast grown on two different carbon sources Mol Biol Cell 1999 10 1859 1872 10359602 10.1091/mbc.10.6.1859 PMC25383

[B15] Smith JJ Marelli M Christmas RH Vizeacoumar FJ Dilworth DJ Ideker T Galitski T Dimitrov K Rachubinsky RA Aitchison JD Transcriptome profiling to identify genes involved in peroxisome assembly and function J Cell Biol 2002 158 259 271 10.1083/jcb.200204059 12135984 PMC2173120

[B16] Kiel JAKW Veenhuis M Klei IJ van der *PEX *genes in fungal genomes: common rare or redundant Traffic 2006 7 1291 1303 10.1111/j.1600-0854.2006.00479.x 16978390

[B17] Gurvitz A Rottensteiner H The biochemistry of oleate induction: transcriptional upregulation and peroxisome proliferation Biochim Biophys Acta 2006 1763 1392 1402 10.1016/j.bbamcr.2006.07.011 16949166

[B18] Smith JJ Ramsey SA Marelli M Marzolf B Hwang D Saleem RA Rachubinski RA Aitchison JD Transcriptional responses to fatty acid are coordinated by combinatorial control Mol Syst Biol 2007 3 115 17551510 10.1038/msb4100157 PMC1911199

[B19] Karpichev IV Durand-Heredia JM Luo Y Small GM Binding characteristics and regulatory mechanisms of the transcription factors controlling oleate-responsive genes in *Saccharomyces cerevisiae* J Biol Chem 2008 283 10264 10275 10.1074/jbc.M708215200 18285336 PMC2447635

[B20] Lin-Cereghino GP Godfrey L de la Cruz BJ Johnson S Khuongsathiene S Tolstorukov I Yan M Lin-Cereghino J Veenhuis M Subramani S Cregg JM Mxr1p, a key regulator of the methanol utilization pathway and peroxisomal genes in *Pichia pastoris* Mol Cell Biol 2007 26 883 897 10.1128/MCB.26.3.883-897.2006 PMC1347016 16428444

[B21] Young ET Dombek KM Tachibana C Ideker T Multiple pathways are coregulated by the protein kinase Snf1 and the transcription factors Adr1 and Cat8 J Biol Chem 2003 278 26146 26158 10.1074/jbc.M301981200 12676948

[B22] Tachibana C Yoo JY Tagne J-B Kacherovsky N Lee TI Young ET Combined global localization analysis and transcriptome data identify genes that are directly coregulated by Adr1 and Cat8 Mol Cell Biol 2005 25 2138 2146 10.1128/MCB.25.6.2138-2146.2005 15743812 PMC1061606

[B23] Leão-Helder NA Krikken AM Klei IJ van der Kiel JAKW Veenhuis M Transcriptional downregulation of peroxisome numbers affects selective peroxisome degradation in *Hansenula polymorpha* J Biol Chem 2003 278 40749 40756 10.1074/jbc.M304029200 12902346

[B24] Ozimek P Lahtchev K Kiel JAKW Veenhuis M Klei IJ van der *Hansenula polymorpha *Swi1 and Snf2 are essential for methanol utilisation FEMS Yeast Res 2004 4 673 682 10.1016/j.femsyr.2004.01.009 15093770

[B25] Rotenberg MO Woolford JL Tripartite upstream promoter element essential for expression of *Saccharomyces cerevisiae *ribosomal protein genes Mol Cell Biol 1986 6 674 687 3023862 10.1128/mcb.6.2.674-687.1986 PMC367559

[B26] Woudt LP Smit AB Mager WH Planta RJ Conserved sequence elements upstream of the gene encoding yeast ribosomal protein L25 are involved in transcription activation EMBO J 1986 5 1037 1040 3013611 10.1002/j.1460-2075.1986.tb04319.x PMC1166898

[B27] Klionsky DJ Cregg JM Dunn WA Jr Emr SD Sakai Y Sandoval IV Sibirny A Subramani S Thumm M Veenhuis M Ohsumi Y A unified nomenclature for yeast autophagy-related genes Dev Cell 2003 5 539 45 10.1016/S1534-5807(03)00296-X 14536056

[B28] Shintani T Huang W-P Stromhaug PE Klionsky DJ Mechanism of cargo selection in the cytoplasm to vacuole targeting pathway Dev Cell 2002 3 825 837 10.1016/S1534-5807(02)00373-8 12479808 PMC2737732

[B29] Monastyrska I Kiel JAKW Krikken AM Komduur JA Veenhuis M Klei IJ van der The *Hansenula polymorpha ATG25 *gene encodes a novel coiled-coil protein that is required for macropexophagy Autophagy 2005 1 92 100 16874036 10.4161/auto.1.2.1832

[B30] Yamashita S-I Yurimoto H Murakami D Yoshikawa M Oku M Sakai Y Lagphase autophagy in the methylotrophic yeast *Pichia pastoris* Genes Cells 2009 14 861 870 10.1111/j.1365-2443.2009.01316.x 19549169

[B31] van Roermund CWT Waterham HR Wanders RJH Fatty acid metabolism in *Saccharomyces cerevisiae* Cell Mol Life Sci 2003 60 1838 1851 10.1007/s00018-003-3076-x 14523547 PMC11138760

[B32] Zwart KD Veenhuis M Plat G Harder W Characterization of glyoxysomes in yeasts and their transformation into peroxisomes in response to changes in environmental conditions Arch Microbiol 1983 136 28 38 10.1007/BF00415606

[B33] Wanders RJA Waterham HR Biochemistry of mammalian peroxisomes revisited Annu Rev Biochem 2006 75 295 332 10.1146/annurev.biochem.74.082803.133329 16756494

[B34] Rodriguez-Manzaneque MT Tamarit J Belli G Ros J Herrero E Grx5 is a mitochondrial glutaredoxin required for the activity of iron/sulfur enzymes Mol Biol Cell 2002 13 1109 1121 10.1091/mbc.01-10-0517 11950925 PMC102255

[B35] Herrero E Ros J Belli G Cabiscol G Redox control and oxidative stress in yeast cells Biochim Biophys Acta 2007 1780 1217 1235 18178164 10.1016/j.bbagen.2007.12.004

[B36] Bener Aksam E de Vries B Klei IJ van der Kiel JAKW Preserving organelle vitality: peroxisomal quality control mechanisms in yeast FEMS Yeast Res 2009 45 1115 1124 10.1111/j.1567-1364.2009.00534.x 19538506

[B37] Goodman JM Maher J Silver PA Pacifico A Sanders D The membrane proteins of the methanol-induced peroxisome of *Candida boidinii*. Initial characterization and generation of monoclonal antibodies J Biol Chem 1986 261 3464 3468 3512558

[B38] Bener Aksam E Jungwirth H Kohlwein SD Ring J Madeo F Veenhuis M Klei IJ van der Absence of the peroxiredoxin Pmp20 causes peroxisomal protein leakage and necrotic cell death Free Radic Biol Med 2008 45 1115 1124 10.1016/j.freeradbiomed.2008.07.010 18694816

[B39] Shalon D Smith SJ Brown PO A DNA microarray system for analyzing complex DNA samples using two-color fluorescent probe hybridization Genome Res 1996 6 639 645 10.1101/gr.6.7.639 8796352

[B40] Gonzalez NA VázquezA A Ortiz Zuazaga HG Sen A Olvera HL Peña de Ortiz S Govind NS Genome-wide expression profiling of the osmoadaptation response of *Debaryomyces hansenii* Yeast 2009 26 111 124 10.1002/yea.1656 19235772

[B41] Graf A Gasser B Dragosits M Sauer M Leparc GG Tüchler T Kreil DP Mattanovich D Novel insights into the unfolded protein response using *Pichia pastoris *specific DNA microarrays BMC Genomics 2008 9 390 10.1186/1471-2164-9-390 18713468 PMC2533675

[B42] Oh KS Kwon O Oh YW Sohn MJ Jung S Kim YK Kim MG Rhee SK Gellissen G Kang HA Fabrication of a partial genome microarray of the methylotrophic yeast *Hansenula polymorpha*: optimization and evaluation of transcript profiling J Microbiol 2004 14 1239 1248

[B43] Park JN Sohn MJ Oh DB Kwon O Rhee SK Hur CG Lee SY Gelissen G Kang HA Identification of the Cadmium-inducible *Hansenula polymorpha SEO1 *gene promoter by transcriptome analysis and its application to whole-cell heavy-metal detection systems Appl Env Microbiol 2007 73 5990 6000 10.1128/AEM.00863-07 PMC2075023 17660305

[B44] Sauer M Branduardi P Gasser B Valli M Maurer M Porro D Mattanovich D Differential gene expression in recombinant *Pichia pastoris *analysed by heterologous DNA microarray hybridisation Microb Cell Fact 2004 3 17 10.1186/1475-2859-3-17 15610561 PMC546231

[B45] Blandin G Llorente B Malpertuy A Wincker P Artiguenave F Dujon B Genomic exploration of the hemiascomycetous yeasts: 13 *Pichia angusta* FEBS Lett 2000 487 76 81 10.1016/S0014-5793(00)02284-5 11152888

[B46] Garcia-Lugo P Gonzalez C Perdomo G Brito N Avila J de la Rosa JM Siverio JM Cloning, sequencing and expression of H.a. YNR1 and H.a. YNI1, encoding nitrate and nitrite reductases in the yeast *Hansenula anomala* Yeast 2000 16 1099 1105 10.1002/1097-0061(20000915)16:12<1099::AID-YEA596>3.0.CO;2-S 10953081

[B47] Avila J Gonzalez C Brito N Siverio JM Clustering of the YNA1 gene encoding a Zn(II)2Cys6 transcriptional factor in the yeast *Hansenula polymorpha *with the nitrate assimilation gene YNT1, YNI1 and YNR1, and its involvement in their transcriptional activation Biochem J 1998 335 647 652 9794807 10.1042/bj3350647 PMC1219828

[B48] Rouillard JM Zuker M Gulari E OligoArray 2.0: design of oligonucleotide probes for DNA microarrays using a thermodynamic approach Nucl Acid Res 2003 31 3057 3062 10.1093/nar/gkg426 PMC162330 12799432

[B49] Gleeson MAG Sudbery PE Genetic analysis in the methylotrophic yeast *Hansenula polymorpha* Yeast 1988 4 293 303 10.1002/yea.320040407

[B50] Van Dijken JP Otto R Harder W Growth of *Hansenula polymorpha *in a methanol-limited chemostat. Physiological responses due to the involvement of methanol oxidase as a key enzyme in methanol metabolism Arch Microbiol 1976 111 137 144 10.1007/BF00446560 1015956

[B51] Waterham HR Keizer-Gunnink I Goodman JM Harder W Veenhuis M Development of multipurpose peroxisomes in Candida Boidinii grown in oleic acid-methanol limited continuous cultures J Bacteriol 1992 174 4057 4063 1350779 10.1128/jb.174.12.4057-4063.1992 PMC206116

[B52] Verduyn C van Dijken JP Scheffers WA Colorimetric alcohol assays with alcohol oxidase J Microbiol Methods 1984 2 15 25 10.1016/0167-7012(84)90027-7

[B53] Cyber-T http://cybert.microarray.ics.uci.edu

[B54] Baldi P Long AD A Bayesian Framework for the analysis of microarray expression data: regularized t-Test and statistical interferences of gene changes Bioinformatics 2001 17 509 519 10.1093/bioinformatics/17.6.509 11395427

[B55] Ecocyc database http://ecocyc.org/

[B56] Van Zutphen T Klei IJ van der Kiel JAKW Pexophagy in *Hansenula polymorpha* Methods Enzymol 2008 451 197 215 full_text 19185722 10.1016/S0076-6879(08)03214-X

